# Burnout among family medicine residents: a cross-sectional nationwide study

**DOI:** 10.1186/s13584-024-00591-2

**Published:** 2024-01-26

**Authors:** Yulia Treister-Goltzman, Tali Samson, Reena Rosenberg, Martine Granek-Catarivas, Anat Gaver, Mordechai Alperin, Aya Biderman

**Affiliations:** 1 Department of Family Medicine and Siaal Research Center for Family Practice and Primary Care, The Haim Doron Division of Community Health, Faculty of Health Sciences, Ben-Gurion University of the Negev, POB 653, 84105 Beer-Sheva, Israel; 2 Clalit Health Services, Southern District, Israel, Beer-Sheva, Israel; 3 Department of Family Medicine, Sackler School of Medicine, Tel Aviv University, Tel Aviv, Israel; 4 Clalit Health Services, Central District, Tel Aviv, Israel; 5 Clalit Health Services, Sharon-Shomron District, Kfar Saba, Israel; 6 Clalit Health Services, Dan-Petach Tikva District, Petach-Tikva, Israel; 7 Department of Family Medicine, The Technion - Israel Institute of Technology, Ruth and Bruce Rappaport Faculty of Medicine, Haifa, Israel; 8 Clalit Health Services, Haifa and Western Galilee District, Haifa, Israel

**Keywords:** Burnout, Work engagement, Psychological flexibility, Work-related stress, Residents

## Abstract

**Background:**

In addition to pressures typical of other medical professions, family physicians face additional challenges such as building long-term relationships with patients, dealing with patients' social problems, and working at a high level of uncertainty. We aimed to assess the rate of burnout and factors associated with it among family medicine residents throughout Israel.

**Methods:**

A cross sectional study based on a self-administered questionnaire.

**Results:**

Ninety family medicine residents throughout Israel completed the questionnaire. The rate of clinically significant burnout, assessed by the composite Shirom-Melamed Burnout Questionnaire score, was 14.4%. In univariate analyses several personal and professional characteristics, as well as all tested psychological characteristics, showed significant associations with burnout. However, in the multivariable logistic regression only psychological work-related characteristics (work engagement, psychological flexibility (reverse scoring), and perceived work-related stress) were significantly associated with burnout at OR (95% CI) = 0.23 (0.06–0.60), 1.31 (1.10–1.71), and 1.16 (1.05–3.749), respectively.

**Conclusion:**

The integration of burnout prevention programs into academic courses during residency could explain the relatively low prevalence of burnout among family medicine residents in this study. Given the strong association of burnout with psychological characteristics, further investment in burnout prevention through targeted structured courses for residents should be encouraged.

**Supplementary Information:**

The online version contains supplementary material available at 10.1186/s13584-024-00591-2.

## Background

Burnout rates among physicians are high, compared to other professions, and even compared to other healthcare specializations [1–3]. High rates of physician burnout appear as early as medical school [4]. A recent meta-analysis found high rates of burnout among residents in different specializations with an average rate of about 50% [5]. A recent comprehensive survey of the Israeli Ministry of Health also demonstrated that, compared to other healthcare sectors, the burnout prevalence was highest among physicians, with residents having the highest burnout rates among them [6]. Previous research in the field has identified three main groups of factors associated with burnout: personal, work-related, and psychological. The last group consists of *work engagement*, which is manifested by energy, dedication and concentration at work, *resilience*, which reflects the individual's ability to adapt and move forward after stressful events, and *psychological flexibility*, which is defined as the ability to be aware of the present moment, and act according to long-term values [7, 8].

In addition to pressures typical of all medical professions, family physicians face additional challenges, such as forming long-term relationships with patients and their families, supporting patients with chronic and fatal diseases, and dealing with patients' social problems. Family physicians operate at a higher level of uncertainty than hospital physicians. The burnout rate among family physicians is 25–60% in different countries [3, 9, 10] and the prevalence also differs among various states within the United States [11].

In Israel, the family medicine residency takes four years. It usually starts with 15 months in one of the accredited primary care clinics under the close supervision of a family physician instructor (stage A). The resident continues with rotations in a hospital: 10 months in internal medicine, five months in pediatrics, two months in the emergency medicine department, two months in psychiatry and two months in elective departments. The resident then returns for the final 12 months at one of the clinics, under the supervision of a family physician instructor (stage B). Family medicine residents are required to pass two exams during their residency, a written level A, and oral level B. Residency programs differ slightly among the family medicine departments in Israel. In some, residents are required to carry out research and work in hospice care. In all, residents attend a weekly university educational program, in which they learn clinical and psycho-social aspects of their profession. As of the time this study was conducted, the total number of family medicine residents in all the HMOs in Israel (“Clalit”, “Maccabi”, “Leumit”, and “Meuhedet”) was around 540, with residents in “Clalit” comprising the vast majority (around 360 residents) (personal communicatication).

The main aims of the present study were to assess burnout rates and factors associated with burnout among family medicine residents throughout Israel. A secondary aim was to characterize the population of family medicine residents in Israel.

## Methods

The study was conducted in five family medicine departments affiliated with the corresponding districts of “Clalit Healthcare Services”, the largest HMO in Israel, which insures more than 52% of the population in Israel. Three of these districts (Central, Dan-Petach-Tikva and Tel-Aviv, and Sharon-Shomron) are in central Israel, one (Haifa and Western Galilee) is in the north, and one (Southern district) is in the southern region of Israel. The study was conducted between January 1, 2022, and September 31, 2022, and statistical analyses were carried out in April 2023.

The study instrument was a self-administered questionnaire that included socio-demographic items, items related to the personal lifestyle of residents, stressful events during the past six months, and items related to the requirements of the residency program (Additional file 1). This questionnaire was formulated for our study after a comprehensive review of the literature on factors associated with burnout among physicians. The questionnaire underwent face validation. An additional part consisted of questionnaires that assessed psychological work-related characteristics of residents. It included the Shirom-Melamed Burnout Questionnaire, a tool to assess burnout (SMBQ). It assesses three dimensions: emotional burnout, physical fatigue, and cognitive exhaustion. The questionnaire was developed in Hebrew in 1992 and the current version was updated in 2006. It contains 16 questions that relate to physical fatigue, cognitive weariness, and emotional exhaustion. A total score ≥ 4.4 was set as the threshold for severe/clinically relevant burnout [12]. The questionnaire is widely used and is considered a valid and reliable tool based on several studies [12, 13]. The Utrecht Work Engagement Scale (UWES-9) was used to assess work engagement. This is a shortened version that has shown good performance compared to a longer version of the same questionnaire. It relates to three main areas of work engagement: vigor, dedication, and absorption. It contains nine questions. A total score ≥ 4.67 indicates a high/very high engagement level and a score ≤ 2.88 indicates low/very low engagement [14]. The questionnaire was translated and validated in Hebrew [15]. The Brief Resilience Scale (BRS) that was developed in 2008, is widely used and is considered valid and reliable. It contains six questions. A total score ≤ 2.99 indicates a low level of resilience and a score ≥ 4.31 indicates a high level [16]. The questionnaire was translated into Hebrew using the backup translation method by the authors of this manuscript. Psychological flexibility was evaluated with the Acceptance and Action Questionnaire 2 (AAQ-2). This questionnaire was developed by Hayes and colleagues in 2004 and the shortened version (seven questions) was developed and validated by Bond and colleagues in 2011 [17]. A total score above 24 is interpreted as “low psychological flexibility” and was found to be related to depression and anxiety. A Hebrew translation, using the backup translation method of the shortened version of this questionnaire, is available [18]. The Primary Care Provider Stress Checklist (PCP-SC) is a structured questionnaire that refers to five areas of stress at work in primary care: communication with patients, managing medical practice, managing administrative work, educational process, relationships with colleagues, and balance between work and “the rest of life”. The questionnaire was developed by Robinson et al. [19] and published in the book “Real behavior change in primary care”. It is very comprehensive for environmental stressors, including an educational aspect, and is therefore suitable for residents. The total score and the score in each of the domains can range from 0—no stress at all, to 100—maximum stress. The questionnaire was translated into Hebrew using the back-translation method and underwent face validation.

### Statistical analysis

To assess the representativeness of our sample, several demographic and residency-related characteristics of the participants were compared to those of the overall population of residents in family medicine of the “Clalit Health Services”, using the two-proportions z-test. Descriptive statistics were conducted to identify the personal, residency-related, and psychological characteristics of the participating residents. Categorical variables such as sex, family status, or country of birth, are presented as frequencies and percentages. Continuous variables, such as age, are described as means and standard deviations and median and range. Categorical variables were tested for differences by the chi-square test/Fisher’s exact test. Differences in continuous variables were identified by the Mann–Whitney rank-sum test. Those independent variables that had a significant association with burnout (*P* < 0.1) were entered into a multivariable logistic regression model.

The sample size was calculated using the WinPepi program based on the following assumptions: (1) in a survey from about eight years ago, the prevalence of burnout among primary care physicians in Israel was 56% (Israel Ministry of Health), and (2) since then family physicians’ responsibilities have increased, the physician shortage in Israel has worsened, and evidence shows that young family medicine physicians have an even higher prevalence of burnout [10]. Therefore, we hypothesized that more than 60% of resident physicians would meet the burnout criteria. At a level of statistical significance of 5%, and power of 80%, at least 90 physicians had to be interviewed to identify this prevalence.

All methods were performed in accordance with relevant guidelines and regulations. The Ethics Committee for Community-based Studies of the Meir Medical Center exempted the study from the requirement to obtain the committee's approval, and the requirement to obtain informed consent.

## Results

Ninety family medicine residents completed the questionnaires. Since the response rate to electronic questionnaires is usually low, the questionnaires were distributed directly by the investigators during study days in five family medicine departments throughout Israel. Only four residents refused to participate. Demographic and residency related characteristics of the participants were compared to those of the overall population of family medicine residents and no statistically significant differences were found.

### Baseline characteristics of the residents

The baseline personal and professional characteristics of the participating residents are presented in Table 1 and Additional file 2. Fifty three (58.9%) of the residents were males, the mean age (± SD) was 34.0 ± 3.6 years. Fifty three (58.9%) were Jews, and 26 (28.9%) were Muslims. Fifty eight (64.4%) stated that they had experienced a stressful event in the past six months, mostly of a personal and family nature (27.6% and 25.9%, respectively). More than 65% did physical activity on a regular or irregular basis, and only 8 (8.9%) smoked. More than 65% had a hobby, the most common being sports and creative activities at N = 25 (27.8%) and N = 15 (16.7%), respectively. Fifty percent graduated a medical school in Israel, 30% studied in Eastern Europe, and 20% in other medical schools in the world. Thirty four (37.8%) were required to do research work and 49 (54.4%) had to participate in home-hospice care.

**Table 1:** Baseline characteristics of study participants (N = 90)

Personal characteristics		Professional and residency-related characteristics	
*Sex, N (%)*		*Years since graduation*	
Males	53 (58.9)	Mean ± SD	5.5 ± 3.6
Females	33 (36.7)	Median	5
Missing	4 (4.4)	Range	0–22
		Missing	4 (4.4)
*Age*		*Seniority as a physician before residency* (years)	
Mean ± SD	34.0 ± 3.6	Mean ± SD	1.38 ± 2.58
Median	34.0	Median	1
Range	27.0–48.0	Range	(0–20)
Missing	2	Missing	6
*Religion, N (%)*		*Country of medical school, N (%)*	
Jew	53 (58.9)	Israel	45 (50.0)
Christian	1 (1.1)	Eastern Europe	27 (30.0)
Muslim	26 (28.9)	Western Europe	6 (6.7)
Other	7 (7.8)	Other	6 (6.7)
Missing	3 (3.3)	Missing	6 (6.7)
*Family status, N (%)*		*Geographical region of residency, N (%)*	
Bachelor	18 (20.0)	Northern	14 (15.6)
Married	63 (70.0)	Central	43 (47.8)
Divorced	1 (1.1)	Southern	29 (32.2)
Live with a permanent partner	4 (4.4)	Missing	4 (4.4)
Missing	4 (4.4)		
*Have children, N (%)*		*Rotation at the present time, N (%)*	
Number of children	52 (57.8)	Clinic A	35 (38.9)
Mean ± SD	1.9 ± 0.8	Internal medicine	12 (13.3)
Median	2	Pediatrics	5 (5.6)
Range	1–5	Elective	12 (13.3)
Children 3 years and younger, N (%)	42 (80.8)	Clinic B	23 (25.6)
Missing	2 (2.2)	Missing	3 (3.3)

The results of work-related psychological characteristics of the participants are presented in Table 2. The score for SMBQ was 3.27 ± 1.29. Thirteen (14.4%) had severe/clinically significant burnout. The score of the physical fatigue subscale was the highest (3.52 ± 1.52), and that of the emotional exhaustion was the lowest (2.84 ± 1.35). Twenty seven (30%) had high scores on at least one of the subscales. The rates of burnout through physical fatigue, cognitive weariness, and emotional exhaustion were 24.4%, 20.0%, and 12.2%, respectively. The score for UWES-9 was 5.13 ± 1.20, indicating overall high work engagement. Sixty (66.7%) met the criteria of high work engagement. The domain with the highest mean score was work dedication (5.39 ± 1.29).

**Table 2:** Personal and work-related psychological characteristics of study participants (N = 90)

Assessed domains and instruments that were used										
Measure	Burnout		Work engagement		Resilience		Psychological flexibility		Workplace stress	
	Shirom-Melamed^a^		Utrecht Work Engagement Scale-9^b^		Brief resilience scale^c^		Acceptance and action questionnaire^d^		Primary Care Provider Stress Checklist^e^	
*Total score*										
Mean ± SD	3.27 ± 1.29		5.13 ± 1.20		3.43 ± 0.72		17.22 ± 7.87		46.28 ± 17.21	
Degrees, N (%)	Severe	13 (14.4)	High	60 (66.7)	High	10 (11.1)	High	76 (84.4)	Not relevant	
	Not severe	77 (85.6)	Moderate Low	26 (28.9) 4 (4.4)	Moderate Low	66 (73.3) 14 (15.6)	Low	14 (15.6)		
*Subscales (if relevant)*										
Mean ± SD	Physical fatigue	3.52 ± 1.52	Vigor	5.08 ± 1.41	Not relevant		Not relevant		Interactions with patients	48.68 ± 18.54
									Practice management	53.59 ± 22.88
	Cognitive weariness	3.31 ± 1.36	Dedication	5.39 ± 1.29						
									Administrative issues	45.77 ± 22.41
									Education/learning	44.60 ± 21.98
	Emotional exhaustion	2.84 ± 1.35	Absorption	4.91 ± 1.24						
									Relationships with colleagues	44.32 ± 19.56
									Balance between work and the "rest of life"	49.23 ± 25.72

^a^The total score ranges from 1 to 7. Total score ≥ 4.4 was the threshold for severe/clinically relevant burnout; ^b^The total score ranges from 1 to 7. A score ≥ 4.67 indicates high/very high engagement and a score ≤ 2.88 indicates low/very low engagement; ^c^The total score ranges from 1 to 5. A total score ≤ 2.99 indicates a low level of resilience and a score ≥ 4.31 indicates a high level; ^d^The total score ranges from 1 to 49. A total score above 24 is interpreted as “low psychological flexibility”, related to depression and anxiety. ^e^The total score and the score in each of the domains can range from 0—no stress at all to 100—maximum stress

The score for BRS was 3.43 ± 0.72, with 66 (73.3%) showing moderate resilience. The psychological flexibility among residents, measured by the AAQ-2, showed a score of 17.22 ± 7.87, with 76 (84.4%) having high psychological flexibility. PCP-SC demonstrated moderate levels of work-related stress, with the higher stress levels in the domains of practice management, relationships with colleagues, and interactions with patients (53.59 ± 22.88, 49.23 ± 25.72, 48.68 ± 18.54, respectively).

### Comparison of residents with and without clinically significant burnout

Additional file 3 shows a comparison of personal and work-related characteristics between residents with and without clinically significant burnout. All the residents with burnout reported a stressful event in the last six months, as compared to 58% of the residents without burnout (*P* = 0.004). Respondents with burnout had more years since graduation (mean 6.2 ± 3.4 vs. 5.4 ± 3.6, *P* < 0.001), most were affiliated with the northern district (61.5% vs. 7.8%, *P* < 0.001), more of them were at the Clinic B stage of their residency (53.8% vs. 20.8%, *P* = 0.04), and they did fewer night shifts per month (mean 1.0 ± 2.0 vs. 2.3 ± 1.9).

**Fig. 1:**
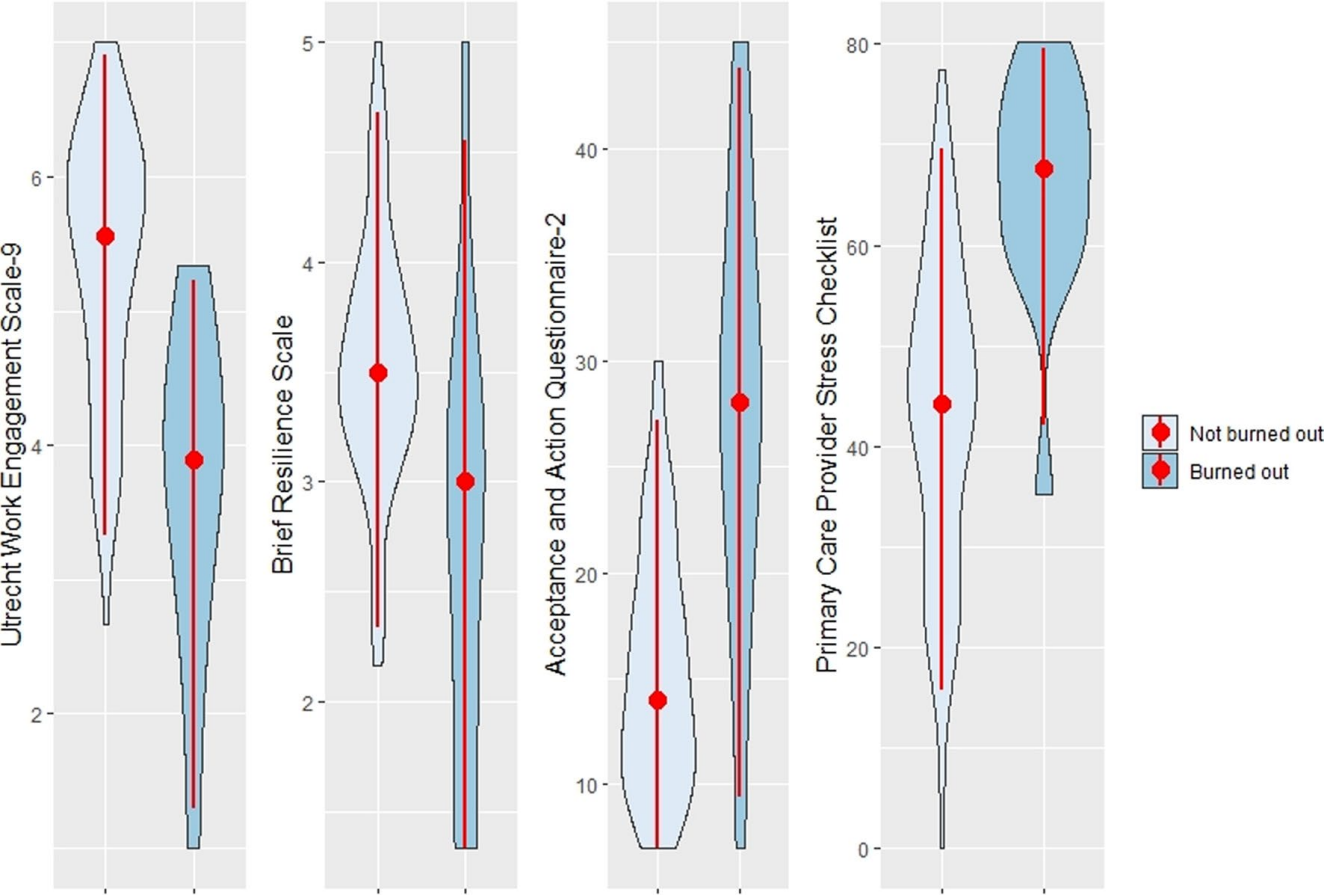
Work-related psychological characteristics of the study participants. Violin plots, depicting the density distribution of participants' scores. Red dots and lines inside each plot indicate the median and 95% confidence interval. Acceptance and Action Questionnaire-2 has a reverse scoring, with higher score indicating lower psychological flexibility. *P* < 0.001 for all psychological tests

Figure 1 presents a comparison of work-related psychological characteristics of residents with and without burnout. The figure consists of violin plots, which are a hybrid of a box plot and a density plot. Residents without burnout showed significantly better psychological characteristics in all psychological domains assessed (median scores of 5.6 vs. 3.9, 3.5 vs. 3.0, 14.0 vs. 28.0, and 44.1 vs. 67.6 for UWES-9, BRS, AAQ-2, and PCP-SC, respectively, *P* < 0.001 for all).

### Multivariable logistic regression model for burnout

Table 3 presents the results of the final logistic regression model. The only significant factors related to burnout were psychological work-related characteristics: work engagement, psychological flexibility, and perceived work-related stress with OR (95% CI) = 0.23 (0.06–0.60), 1.31 (1.10–1.71), and 1.16 (1.05–3.749), respectively). The Hosmer–Lemeshow test (*P* = 1.00) indicated excellent goodness of fit.

**Table 3:** Factors associated with clinically significant burnout among family medicine residents

Variable	OR	95% CI	P-value
Work engagement (Ultrecht-9 questionnaire)	0.23	0.06–0.60	0.010
Psychological flexibility^a^ (Acceptance and Action questionnaire)	1.31	1.10–1.71	0.011
Stress level (Primary care provider stress checklist)	1.16	1.05–1.34	0.013

*OR* odds ratio; *95% CI* 95% confidence interval

^a^The questionnaire of psychological flexibility has a reverse scoring, with higher score indicating lower psychological flexibility

## Discussion

When interpreting the prevalence of burnout in our study, one should note that it was assessed by SMBQ. This questionnaire uses a composite score along three dimensions, emotional exhaustion, cognitive weariness, and physical fatigue and differs conceptually from the most widely used Maslach Burnout Inventory (MBI), which refers to burnout as a multifactorial psychological syndrome. The score in MBI is given separately for each unique dimension, and the person is considered "burned out" if either emotional exhaustion or depersonalization has a high score. We found that according to the composite score of SMBQ, the rate of the clinically significant burnout among family medicine residents in Israel was 14.4%, which is lower than reported in the literature worldwide [3, 9–11] and in Israel [6, 20], including studies among family physicians [21, 22], showing 30–56% burnout rates in different subscales. The use of MBI in those studies could explain the higher rates. In two studies from Ireland [23, 24], in which modified composite overall scoring for MBI was introduced for the first time, burnout rates of 6.6% and 6.8% were found, which are even lower than in our sample. An Israeli study, performed in southern Israel ten years ago among family physicians, found a burnout rate of 9.4% [25]. Burnout in that study was assessed with yet another measure, a subscale of the Compassion Satisfaction and Fatigue Test. The different existing measures of burnout are not completely similar. In addition to the psychological consequences of professional burnout, which are covered by MBI, SMBQ assesses the feeling of physical fatigue. More than that, the emotional exhaustion subscale of MBI was found to correlate more strongly with depression than with other subscales of the questionnaire [26]. The lower rates of burnout found in our study compared to those reported from studies worldwide are at least partly explained by the use of MBI in most of them [3–6, 9, 11, 20–22], with a high score in a single dimension defining a person as “burned out”. Indeed, in our study 30% of the participants had burnout according to at least one of the SMBQ subscales. This prevalence, albeit higher than 14% according to the composite scale, is still among the lowest reported in the world. While the comparison of the prevalence of burnout found in our study with the prevalence found in the studies that used other questionnaires, for the most part MBI, is problematic, it appears that the prevalence of burnout among family medicine residents in Israel is less than reported among physicians worldwide. There are several possible explanations for this finding. The first is that residents in family medicine in Israel have fewer night shifts compared to residents in many other specializations, and less working hours than senior physicians. The second is that, as a part of an academic four-year course for family medicine residents, a program to prevent burnout was set up in some of the departments, such as “Healers’ art” or “Narrative medicine and reflective writing.” In addition, Balint groups are conducted during the 3rd and 4th year of residency [27].

Assessing individual subscales of SMBQ showed higher scores for physical fatigue, than for cognitive weariness and emotional exhaustion. As mentioned above, physical fatigue is not assessed by the MBI questionnaire. Other studies on the topic reported high rates of burnout through emotional exhaustion [11], and depersonalization [23, 28]. The high rates of physical fatigue in our sample can be attributed to the fact that most of the residents were married and parents of young children.

Although in the univariate analysis several personal and professional characteristics, as well as all psychological characteristics showed significant association with burnout, in the multivariable logistic regression only psychological characteristics (work engagement, psychological flexibility and perceived work-related stress) retained a significant association with burnout. The small sample size in our study could have limited the identification of important associations in univariable and, especially, multivariable analyses. Another possible explanation of the result that personal and professional characteristics were no longer statistically significant in the multivariable model is that they were confounders. After controlling for psychological characteristics these associations were no longer statistically significant. Indeed, personal socio-demographic and professional characteristics (such as sex and working hours) were significantly associated with burnout [29] in only few other studies that investigated factors associated with burnout. In contrast, work related psychological characteristics, such as work engagement, psychological flexibility, and perceived work-related stress, were associated strongly with burnout in the vast majority of studies, even after adjustment for other factors [8, 30–32]. Resilience, which had a highly negative correlation with burnout in the univariate analysis, wasn't significant in the multivariable analysis. Other studies, both qualitative and quantitative, demonstrated that this psychological resource played a significant protective role against burnout [33–35]. A possible explanation is that, unlike other studies, we adjusted our model for several psychological factors, and the apparent association between resilience and burnout was due to the confounding effects of other psychological characteristics. While the prevalence of burnout that was found in our study is of local significance, due to the unique residency curriculum and the structure of the Israeli health system, the finding that psychological factors were associated with burnout could certainly be generalized and applied to a broader community of family medicine residents, and physicians in general.

One of the study aims was to describe a population of family medicine residents in Israel. A positive finding was that a relatively high percent of the physicians was engaged in moderate to high frequency physical activities (65.6%). A literature search yielded even higher reported rates of physical activity, up to 92%, among physicians worldwide [36–38]. The smoking rate was low, around 9%. A recent systematic review showed 24% prevalence of smoking among family physicians, much higher than among other medical professions [39]. More than 65% of the respondents had a hobby, which in qualitative studies was a main theme that physicians perceived as important to their well-being [33]. Though these personal characteristics were not associated with burnout, the findings are gratifying, because they contribute to the physical and mental health of young physicians. In the PCP-SC questionnaire, practice management and relationships with colleagues were the main sources of stress. Those are modifiable factors that should inform health care managers and decision makers.

### Study limitations and strengths

One limitation of our study is the relatively small sample. Although it conformed to the sample size calculations based on the study assumptions, it may be small in view of the actual prevalence found in the study.

As previously stated, current literature on burnout among physicians is mostly based on the Maslach burnout instrument. Because of this, it is difficult to compare our findings on burnout rate with other international studies, and a clear conclusion may not be drawn concerning the low burnout rate among family medicine residents in Israel. Furthermore, our findings do not explain the cause of low burnout, nor prove possible explanations we have suggested. Additionally, the cross-sectional design only assessed associations at a single time point, rather than causal or temporal relationships between variables. This could have reduced the ability to identify associations between burnout and some factors in the analyses. Furthermore, as in all questionnaire-based studies, there is a possibility of recall and/or social desirability bias, among others. On the other hand, questionnaires are the most acceptable and feasible way to assess the psychological characteristics of participants. The strengths of this study include the involvement of residents from family medicine departments throughout Israel, and a comprehensive questionnaire that assessed multiple personal, professional, and work-related characteristics, enabling the development of a model with an excellent goodness of fit.

## Conclusion

The prevalence of burnout among family medicine residents in Israel, according to SMBQ, is not very high at around 14%. The integration of burnout prevention programs into academic courses during residency is a possible explanation for this finding. Psychological work-related characteristics, such as high work engagement, high psychological flexibility, and lower perceived work-related stress, but not socio-demographic or professional characteristics, were strong protective factors against burnout. Further investment in burnout prevention through targeted, structured courses for residents should be encouraged.

### Supplementary Information

**Additional file 1.** Questionnaire on the baseline characteristics of the study participants (socio-demographic, personal and residency-specific questions).

**Additional file 2.** Additional baseline characteristics of residents.

**Additional file 3.** Comparison of residents with and without clinically significant burnout.

## Data Availability

The data that support the findings of this study are available from the corresponding author, YTG, upon reasonable request.
