# Supplementary material for: Burnout among family medicine residents: a cross-sectional nationwide study
Source: Isr J Health Policy Res. 2024 Jan 26;13:5. doi: 10.1186/s13584-024-00591-2 (PMC10811917; doi:10.1186/s13584-024-00591-2)
Supplement: Supplementary file 1 — Additional file 1. Questionnaire on the baseline characteristics of the study participants (socio-demographic, personal and residency-specific questions). [file 13584_2024_591_MOESM1_ESM.docx]

Additional file 1. Questionnaire on the baseline characteristics of the study participants (socio-demographic, personal and residency-specific questions)

**1. Birth date:** __________

**2.** **Country of birth:** 1.Israel 2. Western world and South America 3. Former USSR,

**immigration date** (for immigrants) __________

**3. Sex:** 1. Female 2. Male

**4. Religion:** 1. Jew 2. Christian 3. Muslim 4. Other__________

**5. Family status:** 1. Bachelor 2. Married 3. Divorced 4. Live with a permanent partner

**6. Number of children:**__________ **ages of children:**__________

**7. Country of medical school**: 1. Israel 2. Eastern Europe 3. Western Europe 4. Other__________

**8.** **Date of graduation:**__________

**9. Seniority as a physician before residency** (years): __________

**10. Date of the beginning of residency:**__________

**11. Geographical region of residency:** 1. Northern 2. Central 3. Southern

**12. Rotation at the present time:** 1. Clinic A 2. Internal medicine 3. Pediatrics 4. Elective 5. Clinic B

**13. Average number of shifts per months** (for those in the hospital): __________

**14. Did you take level A exam:** 1. Yes 2. No

**15. If "yes", did you pass level A exam?** 1. Yes 2. No

**16. Are you required to do research as part of the residency?** 1. Yes 2. No

**17. Are you required to participate in home-hospice care as part of the residency?** 1. Yes 2. No

**18.** **Have you experienced a stressful event in the last six months** (including happy events such as the birth of a child or moving residence)? 1. Yes. Please circle the type of event: a. Personal b. Family c. Professional d. Other 2.No

**19. Do you exercise?** 1. Yes, regularly (3-4 times a week) 2. Yes, irregularly 3. No

**20. Do you smoke?** 1. Yes 2. No

**21. Do you have a hobby?** 1. Yes. What hobby? __________ 2. No
