# Supplementary material for: Burnout among family medicine residents: a cross-sectional nationwide study
Source: Isr J Health Policy Res. 2024 Jan 26;13:5. doi: 10.1186/s13584-024-00591-2 (PMC10811917; doi:10.1186/s13584-024-00591-2)
Supplement: Supplementary file 2 — Additional file 2. Additional baseline characteristics of residents. [file 13584_2024_591_MOESM2_ESM.docx]

Additional file 2. Additional baseline characteristics of residents

| Personal characteristics | | Professional and residency-related characteristics | |
| --- | --- | --- | --- |
| **Country of birth,** N (%)  Israel  Western world and South  America  Former USSR  Missing | 35 (38.9)  3 (3.3)  50 (55.6)  2 (2.2) | **Number of night shifts per month** (for those in the hospital)  Mean±SD  Median  Range | 2.0±2.0  2  0-4 |
| **Years in Israel** (for those born abroad)  Mean±SD  Median  Range | 25.1±10.5  31  3-34 | **Took Level A exam,** N (%)  Yes  No  Missing  **Passed Level A exam** (for those, who took), N (%)  Yes  No  Missing | 25 (27.8)  62 (68.9)  3 (3.3)  18 (72.0)  6 (24.0)  1 (4.0) |
| **Stressful event in the last six months,** N (%)  Yes  No  Missing  **Type of event** (for those who had one), N (%)  Personal  Family  Professional  Other  Several types  Missing | 58 (64.4)  29 (32.2)  3 (3.3)  16 (27.6)  15 (25.9)  5 (8.6)  4 (6.9)  12 (20.7)  6 (10.3) | **Year in residency**, N (%)  First  Second  Third  Fourth  Missing | 30 (33.3)  21(23.3)  14 (15.6)  19 (21.1)  6 (6.7) |
| **Doing physical activity,** N (%)  Yes, regularly (3-4 times a week)  Yes, irregularly.  No  Missing | 17 (18.9)  42 (46.7)  28 (31.1)  3 (3.3) | **Requirement to do research,** N (%)  Yes  No  Missing | 34 (37.8)  51 (56.7)  5 (5.6) |
| **Smoking,** N (%)  Yes  No  Missing | 8 (8.9)  80 (88.9)  2 (2.2) | **Required to participate in home-hospice care,** N (%)  Yes  No  Missing | 49 (54.4)  37 (41.1)  4 (4.4) |
| **Has a hobby,** N (%)  Yes  No  Missing  **Type of hobby** (for those who have one)**,** N (%)  Sport  Cooking/baking  Reading/learning  new languages  Creative activities  Travel | 59 (65.6)  26 (28.9)  5 (5.6)  25 (27.8)  6 (6.7)  11 (12.2)  15 (16.7)  8 (8.9) |  |  |
