# Supplementary material for: Burnout among family medicine residents: a cross-sectional nationwide study
Source: Isr J Health Policy Res. 2024 Jan 26;13:5. doi: 10.1186/s13584-024-00591-2 (PMC10811917; doi:10.1186/s13584-024-00591-2)
Supplement: Supplementary file 3 — Additional file 3. Comparison of residents with and without clinically significant burnout. [file 13584_2024_591_MOESM3_ESM.docx]

Additional file 3. Comparison of residents with and without clinically significant burnout

| Personal characteristics | | | | Professional and residency-related characteristics | | | |
| --- | --- | --- | --- | --- | --- | --- | --- |
| **Variable** | Without clinically significant burnout  (N=77) | With clinically significant burnout  (N=13) | P-value | **Variable** | Without clinically significant burnout  (N=77) | With clinically significant burnout  (N=13) | P-value |
| **Sex,** N (%)  Males  Females  Missing | 46 (59.7)  27 (35.1)  4 (5.2) | 7 (53.8)  6 (46.2)  0 | 0.55^a^ | **Years since graduation**  Mean±SD  Median  Range  Missing | 5.4±3.6  5  1-22  3 | 6.2±3.4  6  2-12  1 | **<0.001^b^** |
| **Age**  Mean±SD  Median Range  Missing | 34.0±3.4  34.0  27.0-48.0  2 | 34.1±4.3  33.0 29.0-43.0  0 | **<0.001^b^** | **Seniority as a physician before residency** (years)  Mean±SD  Median  Range  Missing | 1.4±2.7  1  0-20  3 | 1.4±2.1  0.5  0-6  3 | **<0.001^b^** |
| **Religion,**  N (%)  Jew  Christian  Muslim  Other  Missing | 47 (61.0)  0  23 (29.9)  4 (5.2)  3 (3.9) | 6 (46.2)  1 (7.7)  3 (23.1)  3 (23.1)  0 | **0.03^a^** | **Country of medical degree**, N (%)  Israel  Eastern Europe  Western Europe  Other  Missing | 38 (49.4)  25 (32.5)  5 (6.5)  4 (5.2)  5 (6.5) | 7 (53.8)  2 (15.4)  1 (7.7)  2 (15.4)  1 (7.7) | 0.27**^a^** |
| **Family status**, N (%)  Bachelor  Married  Divorced  Live with a permanent partner  Missing | 16 (20.8)  54 (70.1)  1 (1.3)  4 (5.2)  2 (2.6) | 2 (15.4) 9 (69.2) 0  0  4 (15.4) | 1.00**^a^** | **Geographical region of residency,** N (%)  Northern  Central  Southern  Missing | 6 (7.8)  40 (51.9)  27 (35.1)  4 (5.2) | 8 (61.5)  3 (23.1)  2 (15.4)  0 | **<0.001^a^** |
| **Have children,** N (%)  Number of children  Mean±SD  Median  Range  Children 3 years and younger, N (%) | 47 (61.0)  1.9±0.8  2  1-5  39 (83.0) | 5 (38.4)  1.8±0.8  2  1-3  3 (60.0) | 0.22**^a^**  **<0.001^b^**  0.51^a^ | **Rotation in the present time,** N (%)  Clinic A  Internal medicine  Pediatrics  Elective  Clinic B  Missing | 32 (41.6)  10 (13.0)  5 (6.5)  11 (14.3)  16 (20.8)  3 (3.9) | 3 (23.1)  2 (15.4)  0  1 (7.7)  7 (53.8)  0 | 0.19**^a^** |
| **Country of birth,** N (%)  Israel  Western world and South America  Former USSR  Missing | 26 (33.8)  3 (3.9)  46 (59.7)  2 (2.6) | 9 (69.2)  4 (30.8) | 0.23**^a^** | **Rotation in the present time,** N (%)  Clinic B  Any other  Missing | 16 (20.8)  58 (75.3)  3 (3.9) | 7 (53.8)  6 (46.2)  0 | **0.04^a^** |
| **Years in Israel** (for born abroad)  Mean±SD  Median  Range | 24.4±11.0  31  3-34 | 30.0±2.8  30  28-32 | **<0.001^b^** | **Number of night shifts** (for those in hospital)  Mean±SD  Median  Range | 2.3±1.9  3.5  0-4 | 1.0±2.0  0  1-4 | **<0.001** |
| **Stressful event in the last six months,** N (%)  Yes  No  Missing  **Type of event** (for those who had), N (%)  Personal  Family  Professional  Other  Several types  Missing | 45 (58.4)  29 (37.7)  3 (3.9)  13 (28.9)  12 (26.7)  5 (11.1)  4 (8.9)  7 (15.6)  4 (8.9) | 13(100.0)  0  0  3 (23.1)  3 (23.1)  0  1 (7.7)  5 (38.5)  1 (7.7) | **0.004^a^** | **Took level A exam,** N (%)  Yes  No  Missing  **Passed level A exam** (for those, who took), N (%)  Yes  No | 18 (23.4)  56 (72.7)  3 (3.9)  15 (83.3)  3 (16.7) | 7 (53.8)  6 (46.2)  0  3 (42.9)  4 (57.1) | **0.045^a^**  0.42**^a^** |
| **Doing physical activity,** N (%)  Yes, regularly (3-4 times a week)  Yeas, irregularly  No  Missing | 14 (18.2)  36 (46.8)  25 (32.4)  2 (2.6) | 3 (23.1)  6 (46.2)  3 (23.1)  1 (7.7) | 0.78**^a^** | **Year in residency**, N (%)  First  Second  Third  Fourth  Missing | 28 (36.4)  18 (23.4)  12 (15.6)  14 (18.2)  5 (6.5) | 2 (15.4)  3 (23.1)  2 (15.4)  5 (38.5)  1 (7.7) | 0.30**^a^** |
| **Smoking,** N (%)  Yes  No  Missing | 6 (7.8)  69 (89.6)  2 (2.6) | 2 (15.4)  11 (84.6)  0 | 0.34**^a^** | **Requirement to do research,** N (%)  Yes  No  Missing | 30 (39.0)  42 (54.5)  5 (6.5) | 4 (30.8)  9 (69.2) | 0.55**^a^** |
| **Having a hobby,** N (%)  Yes  No  Missing  **Type of hobby** (for those who have)**,** N (%)  Sport  Cooking/baking  Reading/learning new languages  Creative works  Travelling | 52 (67.5)  20 (26.0)  5 (6.5)  22 (42.3)  6 (11.5)  10 (19.2)  11 (21.2)  7 (13.5) | 7 (15.4)  6 (84.6)  0  3 (23.1)  0  1 (7.7)  4 (30.8)  1 (7.7) | 0.20**^a^**  0.64**^a^** | **Requirement to participate in home- hospice care,** N (%)  Yes  No  Missing | 42 (54.5)  31 (40.3)  4 (5.2) | 7 (53.8)  6 (46.2) | 1.00**^a^** |
